# Experimental Demonstration of Long-Range Underwater Acoustic Communication Using a Vertical Sensor Array

**DOI:** 10.3390/s17071516

**Published:** 2017-06-27

**Authors:** Anbang Zhao, Caigao Zeng, Juan Hui, Lin Ma, Xuejie Bi

**Affiliations:** 1 Acoustic Science and Technology Laboratory, Harbin Engineering University, Harbin 150001, China; zhaoanbang@hrbeu.edu.cn (A.Z.); cgzeng@hrbeu.edu.cn (C.Z.); malin@hrbeu.edu.cn (L.M.); bixuejie@hrbeu.edu.cn (X.B.); 2 College of Underwater Acoustic Engineering, Harbin Engineering University, Harbin 150001, China; 3 National Key Laboratory of Science and Technology on Underwater Acoustic Antagonizing, China State Shipbuilding Corporation Systems Engineering Research Institute, Beijing 100036, China

**Keywords:** long-range UWA communication, shallow water, vertical sensor array, composite channel, virtual time reversal mirror

## Abstract

This paper proposes a composite channel virtual time reversal mirror (CCVTRM) for vertical sensor array (VSA) processing and applies it to long-range underwater acoustic (UWA) communication in shallow water. Because of weak signal-to-noise ratio (SNR), it is unable to accurately estimate the channel impulse response of each sensor of the VSA, thus the traditional passive time reversal mirror (PTRM) cannot perform well in long-range UWA communication in shallow water. However, CCVTRM only needs to estimate the composite channel of the VSA to accomplish time reversal mirror (TRM), which can effectively mitigate the inter-symbol interference (ISI) and reduce the bit error rate (BER). In addition, the calculation of CCVTRM is simpler than traditional PTRM. An UWA communication experiment using a VSA of 12 sensors was conducted in the South China Sea. The experiment achieves a very low BER communication at communication rate of 66.7 bit/s over an 80 km range. The results of the sea trial demonstrate that CCVTRM is feasible and can be applied to long-range UWA communication in shallow water.

## 1. Introduction

Due to their multipath interactions, UWA channels are considered one of the most challenging wireless communication environments. The slow sound speed, the sound absorption and the variability of water can produce serious interference in UWA signals. Especially in shallow water channel [1], specific multipath interference is strong enough to cause severe ISI resulting in symbol errors in UWA communication.

TRM can be used for time compression and spatial focusing, making channel equalization and multipath interference reduction come true, in the absence of any prior knowledge [2], so it is widely studied in the field of UWA communications. Dowling first suggested active time reversal mirror (ATRM) [3], made definitions of its application in UWA field and did some basic theoretical analysis. However, ATRM requires two-way transmission by using a source-receive array (SRA) and a VSA, which makes it relatively complex. Each sensor of the SRA is required to both receive and transmit signals, and the signals need to be transmitted twice in the acoustic channel, resulting in a considerable lapse of time and low efficiency. In addition, two-way transmission increases the extra noise and causes the signal amplitude to fade twice. In view of the complexity of ATRM, PTRM is put forward in [4]. Compared with ATRM, PTRM requires only a VSA and one-way transmission. In [5,6,7,8], some theoretical derivations of TRM technology of different array shapes are made. The research group led by Kuperman verified the principles of TRM through a sea trial [2] in April 1996. Their work promoted the application of TRM in UWA communication. In [9,10,11,12], ATRM technology is applied to UWA communication. References [13,14,15,16] studied UWA communication using PTRM, and pointed out that PTRM can realize adaptive channel equalization, suppress the ISI and thereby improve the performance of UWA communication systems. In practice, there is always some residual ISI after time reversal processing. In [17,18,19], PTRM was followed by a single-channel decision-feedback equalizer (DFE) to eliminate the residual ISI, and it was confirmed that PTRM combined with a DFE (PTRM-DFE) can improve the performance significantly over PTRM. In [20,21,22], TRM was applied to multiuser or multiple-input-multiple-output (MIMO) communications due to the spatial focusing capability. Recently, with the advances in algorithm development, there is ample scope for research to be done in TRM-based communications [23].

In summary, ATRM is both expensive and difficult to implement, and is not practical, while PTRM can obtain the same theoretical performance in a simple way. Nevertheless, PTRM needs to estimate the channel impulse response of each sensor of the VSA, which is somewhat unrealistic in complex long-range shallow water environments, because the SNR of the received signals of each sensor is too weak to estimate the channels accurately. Therefore, this paper proposes a CCVTRM technology for UWA communication and makes some theoretical derivations of this technology. The proposed method utilizes the spatial gain of the VSA to estimate the single composite channel, thus implementing the CCVTRM and achieving the temporal focusing. A long-range communication experiment in shallow water conducted in the South China Sea validates the practicability of this technology.

## 2. Theory of UWA Communication Using CCVTRM

### 2.1. Principles of Signal Coding

**Figure 1:**
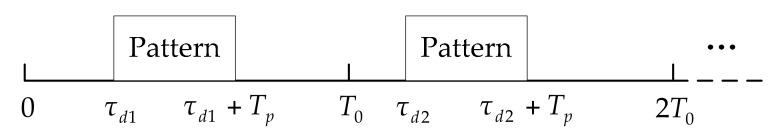
Pattern time delay shift coding scheme.

Pattern time delay shift coding scheme [24,25] is a kind of pulse position coding scheme. It modulates the digital information in the time delay shift value of the pattern, rather than in the code waveform. And different time delay shift values represent different information. Figure 1 shows the scheme of pattern time delay shift coding.

As shown in Figure 1, $\tau_{\mathrm{di}}(i=1,2,3,\ldots )$ is the time delay shift value, means the position of pattern in the encoding time window. $T_{p}$ is the pattern pulse width and *T*_0_ denotes the length of one symbol. The encoding time window for information coding is $T_{c}=T_{0}-T_{p}$. The quantization unit of time delay shift is $\Delta \tau =T_{c}/(2^{n}-1)$ if one symbol carries n bits digital information, thereby the time delay shift value is $\tau_{d}=k\cdot \Delta \tau$, where $k=1,2,\ldots ,2^{n}-1$. The duty ratio is $\eta =T_{p}/T_{0}$, whose value is smaller than 1. It means this scheme can save the power during the communication, which is very valuable for UWA communication system.

### 2.2. Principles of CCVTRM

**Figure 2:**
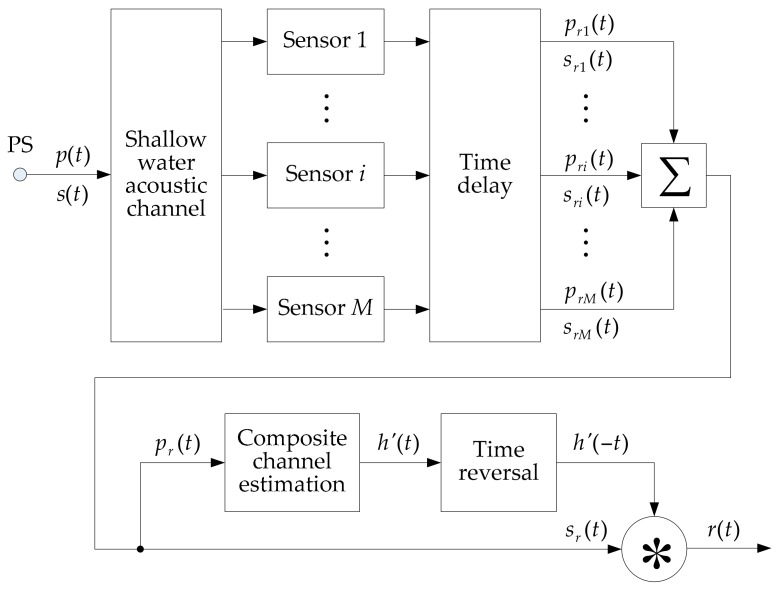
The procedure of CCVTRM.

In a long-range shallow water environment, it is always impossible to estimate the channel pulse response of each sensor to implement traditional PTRM because of the low SNR. Although the array signal processing (ASP) can achieve spatial gain, the ISI resulting from multipath propagation in shallow water cannot be suppressed. In view of this, CCVTRM is put forward to improve the VSA processing. Figure 2 illustrates the procedure of CCVTRM.

The point source (PS) transmits a probe pulse signal p(t) first, and then sends the data stream s(t). After the signals pass through the shallow water acoustic channel, they are time delay processed, so that the direct sounds of each sensor are thought to be received at the same time. Signal propagation through a shallow water acoustic channel is characterized by the channel impulse response function. And assuming that the ocean channel keeps invariable between the transmission of the probe signal and the reception of the data stream, so the received probe signal and data stream on the number i sensor can be expressed as:

(1) $$p_{\mathrm{ri}}(t)=p(t)*h_{i}(t)+n_{\mathrm{pi}}(t)$$

(2) $$s_{\mathrm{ri}}(t)=s(t)*h_{i}(t)+n_{\mathrm{si}}(t)$$

where ‘∗’ denotes convolution, $h_{i}(t)$ is the channel impulse response, and $n_{\mathrm{pi}}(t)$ and $n_{\mathrm{si}}(t)$ are ocean noises. Due to the large multipath spreads, the channel impulse response $h_{i}(t)$ can be expressed as:

(3) $$h_{i}(t)=\sum_{j=1}^{N_{i}}a_{\mathrm{ij}}\delta (t-\tau_{\mathrm{ij}})$$

where $N_{i}$ is the number of multipath between the source and the number i receiver, and $a_{\mathrm{ij}}$ and $\tau_{\mathrm{ij}}$ are the amplitude and time delay of the number j path.

During the long-range UWA communication in shallow water, the SNR of received signals of each receiver is so low that $h_{i}(t)$ cannot be estimated, so that the traditional PTRM cannot be realized. Therefore, ASP is necessary. Here, the conventional beam forming (CBF) is used, including time delay processing and uniform weighted summation. After the time delay processing, it is thought that the transmission time of the signal in the corresponding sub-channel of each receiver is the same, and suppose $\tau_{i1}$ of each sub-channel equal to $\tau_{1}$. So the outputs of the VSA can be expressed as:

(4) $$\begin{array}{cc} p_{r}(t) & =\sum_{i=1}^{M}p_{\mathrm{ri}}(t)\\ & =p(t)*h(t)+\sum_{i=1}^{M}n_{\mathrm{pi}}(t) \end{array}$$

(5) $$\begin{array}{cc} s_{r}(t) & =\sum_{i=1}^{M}s_{\mathrm{ri}}(t)\\ & =s(t)*h(t)+\sum_{i=1}^{M}n_{\mathrm{si}}(t) \end{array}$$

where:

(6) $$h(t)=\sum_{i=1}^{M}h_{i}(t)$$

Combining Equations (3) and (6), the composite channel can be expressed as:

(7) $$\begin{array}{cc} h(t) & =\sum_{i=1}^{M}\sum_{j=1}^{N_{i}}a_{\mathrm{ij}}\delta (t-\tau_{\mathrm{ij}})\\ & =\sum_{i=1}^{M}a_{i1}\delta (t-\tau_{1})+\sum_{i=1}^{M}\sum_{j=2}^{N_{i}}a_{\mathrm{ij}}\delta (t-\tau_{\mathrm{ij}})\\ & =A_{1}\delta (t-\tau_{1})+\sum_{k=2}^{N}A_{k}\delta (t-\tau_{k}) \end{array}$$

where $N=\sum_{i=1}^{M}N_{i}-M+1$, $A_{1}=\sum_{i=1}^{M}a_{i1}$, and when k≥2, $A_{k}$ and $\tau_{k}$ correspond to $a_{\mathrm{ij}}$ and $\tau_{\mathrm{ij}}$, respectively.

The function h(t), called the composite channel of the VSA, is considered to be a virtual ocean acoustic channel which the signals propagate through. This composite channel is also a very complex channel as well as the actual UWA channel.

ASP makes the direct sounds of each sensor coherently superimposed and provides a higher SNR to the output signals $p_{r}(t)$ and $s_{r}(t)$, benefitting from the spatial gain of the VSA. However, because of the existence of the composite channel, the output data stream is still affected by the multipath extension. Hence, through the copy-correlation processing of $p_{r}(t)$, the composite channel estimation denoted as $h^{\prime }(t)$ is output. The final data stream is the convolution of $s_{r}(t)$ with the time-reversed composite channel estimation $h^{\prime }(-t)$, which can be expressed as:

(8) $$\begin{array}{cc} r(t) & =s_{r}(t)*h^{\prime }(-t)\\ & =s(t)*h(t)*h^{\prime }(-t)+\sum_{i=1}^{M}n_{\mathrm{si}}(t)*h^{\prime }(-t)\\ & =s(t)*\hat{h}(t)+\sum_{i=1}^{M}n_{\mathrm{si}}(t)*h^{\prime }(-t) \end{array}$$

where:

(9) $$\hat{h}(t)=h(t)*h^{\prime }(-t)$$

$\hat{h}(t)$ called virtual channel, considered to be the exclusive channel the data stream propagates through, is the correlation of the composite channel h(t) and its estimation $h^{\prime }(t)$. If the estimation $h^{\prime }(t)$ is approximated as the composite channel h(t), the virtual channel $\hat{h}(t)$ will tend to be a delta function with a high correlation peak. Moreover, as the complexity of h(t) increases, the virtual channel $\hat{h}(t)$ is closer to a delta function. Ideally, the virtual channel $\hat{h}(t)$ can be thought of as a simple channel only with a single path. That is, CCVTRM can refocus the energy of the dispersed signal caused by multipath.

To calculate the output SNR gain of CCVTRM, given that the SNR of each path signal is equal and noises are uncorrelated in the array elements, we can obtain:

(10) $$\begin{array}{cc} s_{r}(t) & =s(t)\times h(t)+n(t)\\ & =\sum_{k=1}^{N}\left[A_{k}s(t-\tau_{k})+n_{k}(t)\right] \end{array}$$

(11) $$E[n_{k}(t)n_{l}(t)]=0,\;k\neq l$$

(12) $${\mathrm{SNR}}_{1}=\frac{A_{1}^{2}}{\sigma_{1}^{2}}=\ldots =\frac{A_{k}^{2}}{\sigma_{k}^{2}}=\ldots =\frac{A_{N}^{2}}{\sigma_{N}^{2}}$$

where $n_{k}(t)$ denotes the noise of the number k path and $\sigma_{k}^{2}$ denotes the noise variance, and ‘E[⋅]’ denotes expected value. After going through CCVTRM, the final data stream can be rewritten as:

(13) $$r(t)=\sum_{k=1}^{N}A_{k}s(t)+\sum_{k=1}^{N}n_{k}(t)$$

The output SNR is:

(14) $${\mathrm{SNR}}_{2}={\left(\sum_{k=1}^{N}A_{k}\right)}^{2}/\left(\sum_{k=1}^{N}\sigma_{k}^{2}\right)$$

Combining Equations (12) and (14), we can obtain:

(15) $${\mathrm{SNR}}_{2}=\frac{A_{1}^{2}}{\sigma_{1}^{2}}\left[1+\left(\sum_{k=1}^{N}\sum_{\begin{array}{l} l=1\\ l\neq k \end{array}}^{N}A_{k}A_{l}\right)/\left(\sum_{k=1}^{N}A_{k}^{2}\right)\right]$$

Thus, the SNR gain of CCVTRM is:

(16) $$G_{\mathrm{VTRM}}=10\log_{10}\left[1+\left(\sum_{k=1}^{N}\sum_{\begin{array}{l} l=1\\ l\neq k \end{array}}^{N}A_{k}A_{l}\right)/\left(\sum_{k=1}^{N}A_{k}^{2}\right)\right]$$

As we can see in Equation (16), the more complex the multipath, the higher SNR gain of CCVTRM we can obtain. In a word, CCVTRM for the VSA processing not only has a simple calculation, but also gets the spatial gain from the VSA and the temporal focusing gain from TRM, moreover, it can mitigate the ISI caused by multipath.

### 2.3. Principles of Detection and Channel Estimation

The key to implementing CCVTRM is detecting the probe pulse signal and estimating the composite channel. From the signal processing point of view, copy-correlation can be viewed as a matched filter that can achieve a maximum SNR under ideal conditions. In this paper, copy-correlation technology is used to detect the probe signal and estimate the composite channel.

In the absence of multipath, the output probe signal of the VSA can be expressed as:

(17) $$p_{r}(t)=A_{1}p(t-\tau_{1})+n_{p}(t)$$

where $n_{p}(t)=\sum_{i=1}^{M}n_{\mathrm{pi}}(t)$.

After copy-correlation processing, the output is:

(18) $$\begin{array}{cc} R(t) & =\int p_{r}(\tau )p(\tau -t)d\tau \\ & =A_{1}\int p(\tau -\tau_{1})p(\tau -t)d\tau +n_{p}^{\prime }(t) \end{array}$$

where $n_{p}^{\prime }(t)=\int n_{p}(\tau )p(\tau -t)d\tau$, denotes the noise component of the output.

Because the probe pulse signal and the noise are uncorrelated, the noise component is weak. Consequently, a correlation peak can be easily found at $t=\tau_{1}$, which means the probe pulse signal is detected.

Taking multipath into account, Equations (17) and (18) can be modified as:

(19) $$p_{r}(t)=\sum_{k=1}^{N}A_{k}p(t-\tau_{k})+n_{p}(t)$$

(20) $$\begin{array}{cc} R(t) & =\int p_{r}(\tau )p(\tau -t)d\tau \\ & =\int \left[\sum_{k=1}^{N}A_{k}p(\tau -\tau_{k})\right]p(\tau -t)d\tau +\int n_{p}(\tau )p(\tau -t)d\tau \\ & =\sum_{k=1}^{N}A_{k}\int p(\tau -\tau_{k})p(\tau -t)d\tau +n_{p}^{\prime }(t) \end{array}$$

It can be seen that the output of copy-correlation contains multiple correlation peaks and the positions of the peaks corresponding to time delay of the multiple paths. Considering the effects of noises, we set a threshold and keep the peaks that higher than the threshold. Then the channel impulse response is obtained.

## 3. Sea Experiment

**Figure 3:**
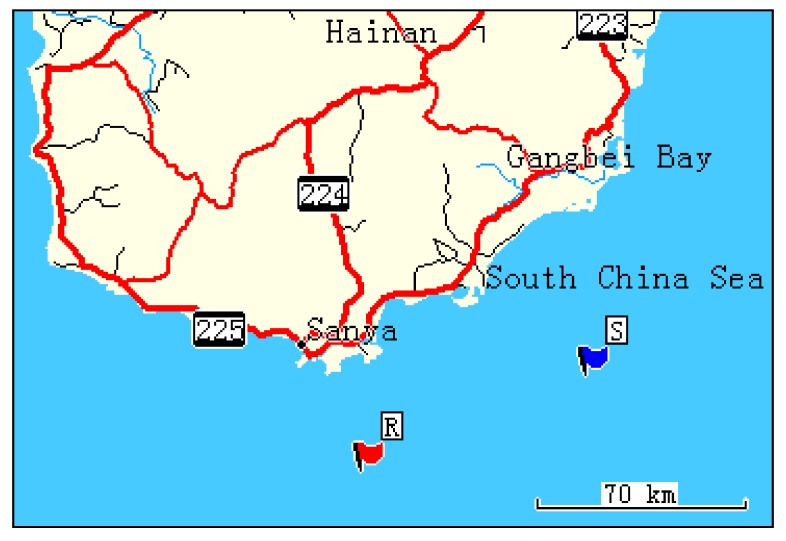
Sea experiment area and the vessels’ locations.

A long-range UWA communication experiment using CCVTRM was conducted in the South China Sea. Figure 3 illustrates the experiment area and the vessels’ locations. The vessel which has a single sound source for transmitting information moored at the point S, while the vessel that deployed the VSA anchored at the point R. Table 1 shows the coordinates of the point S and R.

**Figure 4:**
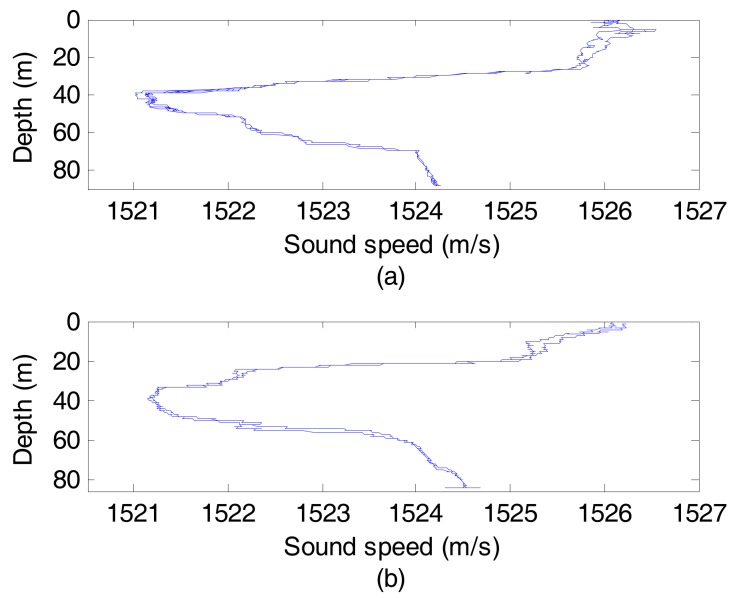
Sound speed profiles of the test points. (**a**) The point S; (**b**) The point R.

**Figure 5:**
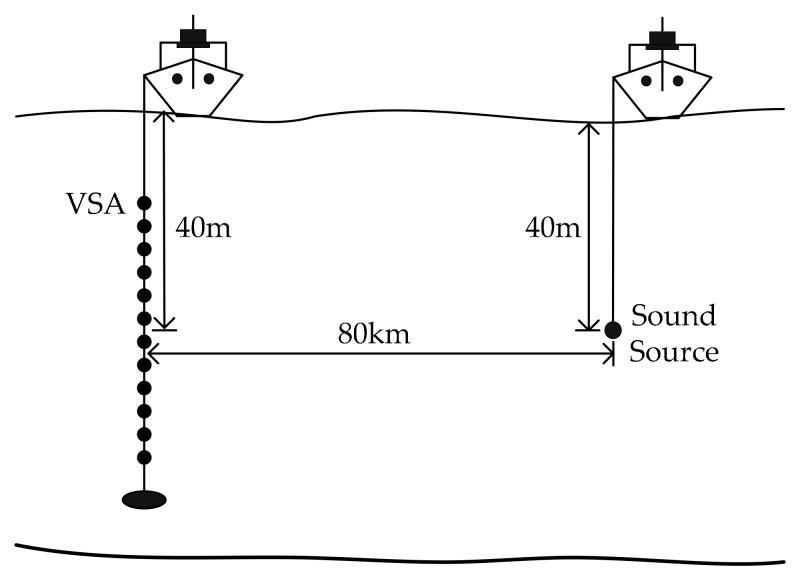
Schematic of the UWA communication experiment.

Figure 4 shows the sound speed profiles of the point S and R. It can be seen that the inflexion points of the sound speed profiles are about 40 m deep. Figure 5 is a schematic of the experiment. Considering the sound speed profiles, the sound source, operated at a 300 Hz bandwidth (500–800 Hz) with a source level of 186 dB, was setup at 40 m deep. Likewise, the VSA located 80 km far away from the sound source was deployed with its acoustic center at the same depth. The VSA is composed of 12 sensors with an equal spacing of 1.5 m.

The procedure started with sending a probe signal to estimate the composite channel of the VSA, followed by the data stream. Both the probe signal and the data stream were using LFM signal form with a bandwidth of 500–800 Hz. And the sampling rate was 48 kHz at both the transmitter end and the receiver end. Trials at different communication rates were conducted by adjusting the pattern pulse width and the length of the encoding time window. Table 2 indicates the detailed values of $T_{p}$, $T_{c}$ and the corresponding communication rate. A communication experiment with a communication rate of 50 bit/s is taken as an example to describe the signal processing.

**Figure 6:**
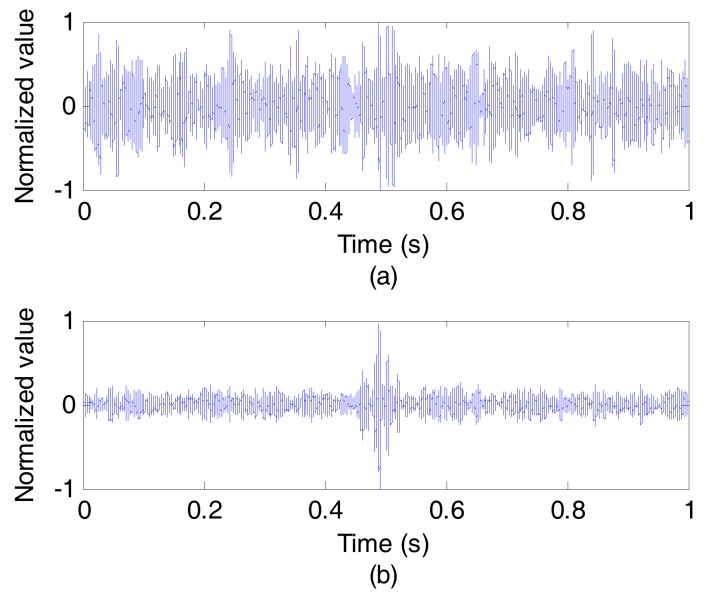
The detection results of the probe pulse signal. (**a**) Before ASP; (**b**) After ASP.

Figure 6 shows the very different detection results of the probe pulse signal before and after ASP, wherein Figure 6a shows the detection result of a single sensor, while Figure 6b is the detection result after ASP. Because of the long propagation distance, the SNR of the signal received by a single sensor is so low that the probe pulse signal can not be detected as shown in Figure 6a, and thus the channel impulse response of the single sensor can not be estimated. However, ASP brings the probe pulse signal a higher SNR, so that the probe pulse signal after ASP can be easily detected as shown in Figure 6b.

**Figure 7:**
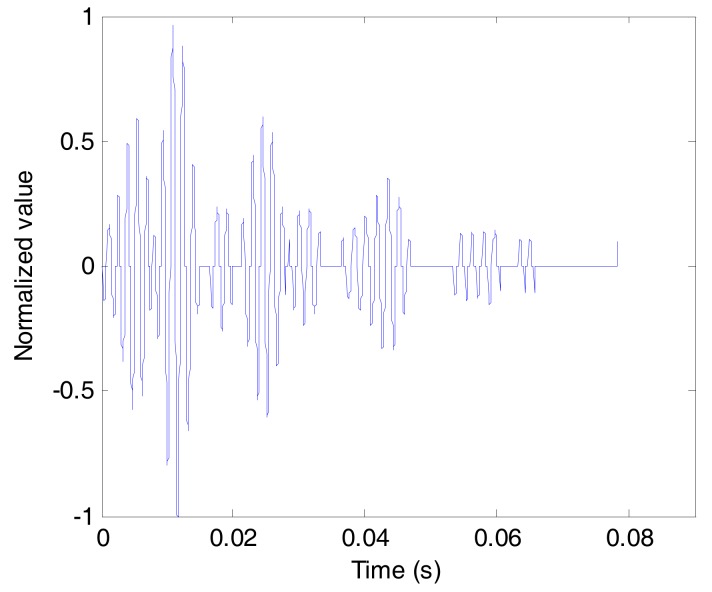
The estimation of the composite channel.

**Figure 8:**
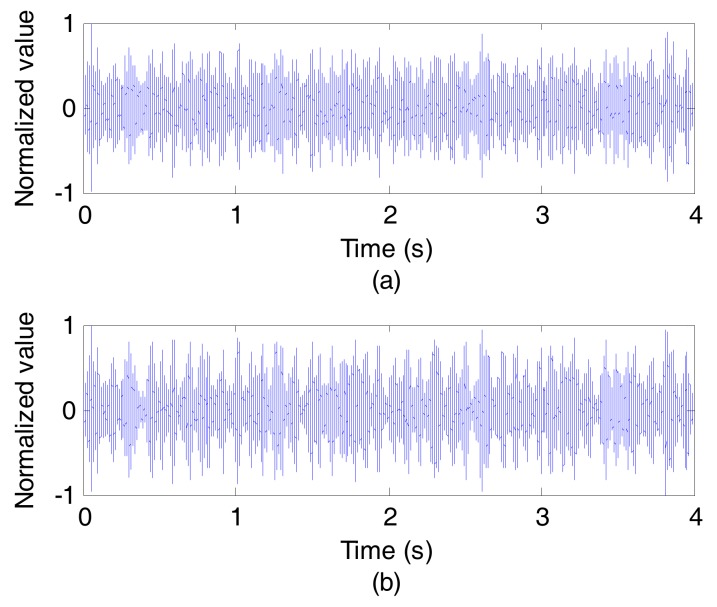
The waveforms of the data stream. (**a**) NO CCVTRM; (**b**) CCVTRM. Note: NO CCVTRM means only using ASP; CCVTRM means using CCVTRM after ASP.

As can be seen in Figure 7, the composite channel estimation, measured by the probe pulse signal, has a very complex multipath structure and a wide time delay spread nearly 80 ms. As a result, the symbols in the data stream are broadened, leading to strong ISI from temporal overlap, which is bad for UWA communication. Thus, using CCVTRM to process the data stream after ASP is necessary. Figure 8 illustrates the waveforms of the data stream before and after CCVTRM. Comparing Figure 8a and Figure 8b, it is clear that CCVTRM can compress the signal in the time domain, thus focusing the energy of the signal and mitigating the ISI.

**Figure 9:**
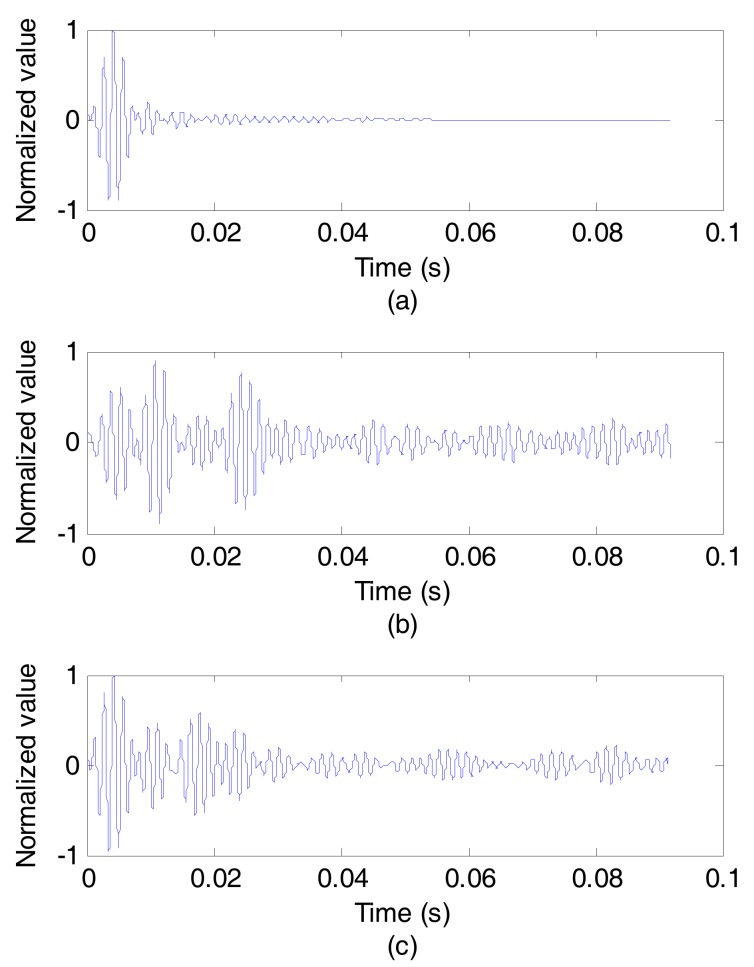
The outputs of the synchronous detection. (**a**) Original; (**b**) NO CCVTRM; (**c**) CCVTRM.

**Figure 10:**
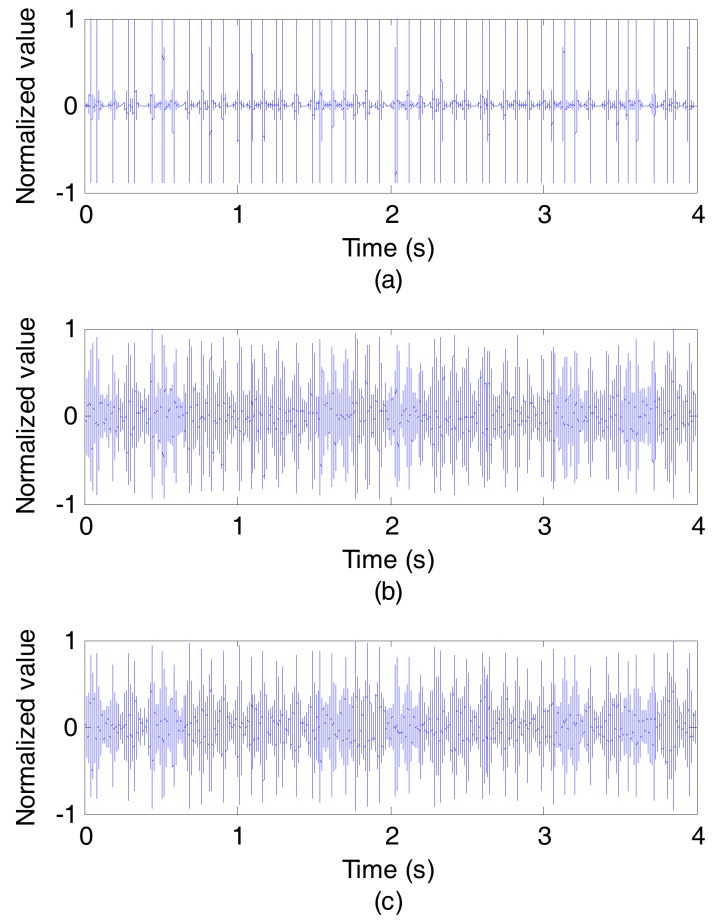
The decoded results. (**a**) Original; (**b**) NO CCVTRM; (**c**) CCVTRM.

**Figure 11:**
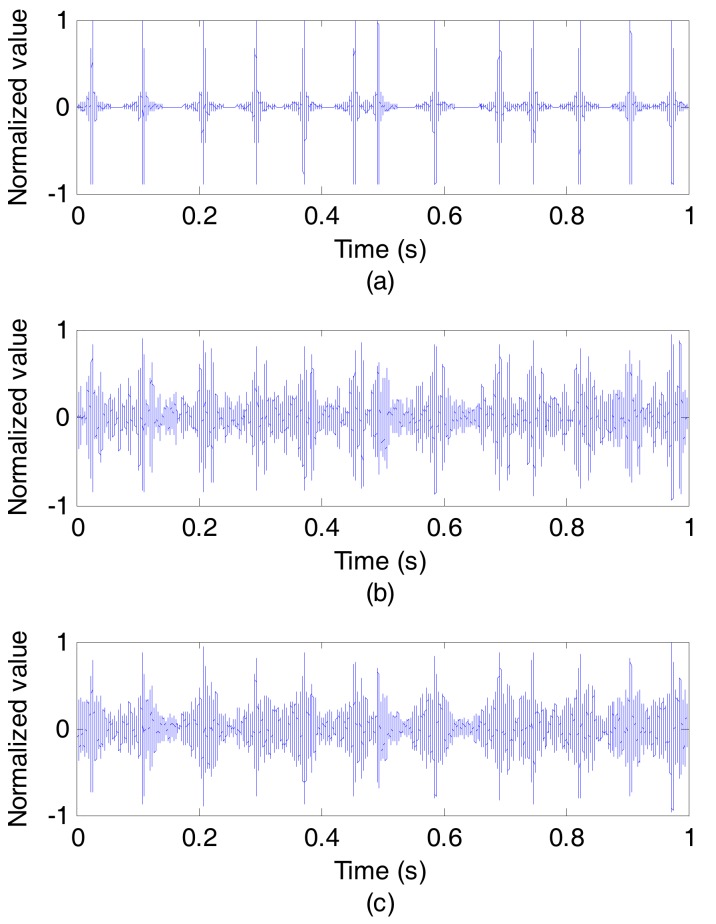
A partial enlargement of decoded results. (**a**) Original; (**b**) NO CCVTRM; (**c**) CCVTRM.

Figure 9 and Figure 10 show the results of the experiment at communication rate of 50 bit/s. Figure 9a depicts the outputs of the synchronous detection of the original signal sent from sound source, while Figure 9b without using CCVTRM and Figure 9c using CCVTRM. Obviously, some pseudo correlation peaks appearing in Figure 9b will confuse the judgment of the sync header’s position. On the contrary, the correlation peak can be easily found and the position of sync signal is complete clear after using CCVTRM, as shown in Figure 9c and Figure 10 illustrates the decoded results, including Figure 10a of the original signal, Figure 10b without using CCVTRM and Figure 10c using CCVTRM. Figure 11 is a partial enlargement of decoded results shown in Figure 10. Indeed, the application of CCVTRM makes the decoding correlation peak of each symbol become single and easy to determine, consequently, reducing the BER.

Since UWA channel is rapidly time-varying, the duration of signal frame should be short. In this case, we transmitted 196 bits of information data in each communication trial and changed the communication rates. Table 3 shows the decoded results of UWA communication trials at different communication rates. The results indicate that the performance of UWA communication is dramatically improved thanks to the CCVTRM processing. It should be pointed out that zero BER means there is no error in the statistical sample, that is to say, the actual BER is very low. In order to verify the stability of the CCVTRM communication scheme, the trials described above were all repeated seven times. Table 4 shows all the decoded results of UWA communication trials using CCVTRM. It is obvious that CCVTRM communication scheme performs well and this scheme achieves a 66.7 bit/s communication rate at a very low BER under a 300 Hz bandwidth, indicating that CCVTRM technology is robust.

## 4. Conclusions

In view of the complex environment of long-range UWA communication in shallow water, this paper proposes a CCVTRM for the VSA processing to improve the performance of UWA communication. Using the VSA can obtain a certain spatial gain and enhance the SNR of received signals, which is good for the UWA signal processing. As well as the general ocean acoustic channels, the composite channel of the VSA has complex multipath structure and wide time delay spread, resulting in severe ISI and high BER. For the array processing, CCVTRM can effectively reform the multipath structure of the composite channel and get the energy focusing gain, so as to mitigate the ISI and reduce the BER. The UWA communication experiment achieves a low BER communication at communication rate of 66.7 bit/s over an 80 km range, demonstrating the practicability and robustness of CCVTRM. It turns out that this technology can be applied to long-range UWA communication in shallow water.

## Figures and Tables

**Table 1:** The coordinates of the test points.

The Test Point	Latitude	Longitude
S	18°12′48″ N	110°25′16″ E
R	17°55′25″ N	109°41′58″ E

**Table 2:** The values of $T_{p}$, $T_{c}$ and communication rate.

*T_p_* (ms)	*T_c_* (ms)	Communication Rate (bit/s)
8	12	200
16	24	100
24	36	66.7
32	48	50
40	60	40
80	120	20

**Table 3:** The decoded results at different communication rates.

Communication Rate (bit/s)	BER(%)	
	NO CCVTRM	CCVTRM
200	42.35	16.84
100	21.43	3.57
66.7	15.31	0
50	7.65	0
40	4.08	0
20	1.53	0

**Table 4:** The decoded results of UWA communication trials using CCVTRM.

Communication Rate (bit/s)	BER(%)						
	1	2	3	4	5	6	7
200	16.84	25.00	17.35	16.33	15.82	17.86	21.43
100	3.57	5.61	1.53	2.04	3.57	4.08	4.59
66.7	0	0	0.51	0	0	0	0
50	0	0	0	0	0	0	0
40	0	0	0	0	0	0	0
20	0	0	0	0	0	0	0
